# Pd@Py_2_PZ@MSN as a Novel and Efficient Catalyst for C–C Bond Formation Reactions

**DOI:** 10.3389/fchem.2022.838294

**Published:** 2022-03-31

**Authors:** Mohammad Hosein Sayahi, Mansoureh Toosibashi, Mehdi Bahmaei, Hosein Lijan, Leila Ma'Mani, Mohammad Mahdavi, Saeed Bahadorikhalili

**Affiliations:** ^1^ Department of Chemistry, Payame Noor University (PNU), Tehran, Iran; ^2^ School of Chemistry, College of Science, University of Tehran, Tehran, Iran; ^3^ Department of Nanotechnology, Agricultural Biotechnology Research Institute of Iran (ABRII), Agricultural Research Education and Extension Organization (AREEO), Karaj, Iran; ^4^ Endocrinology and Metabolism Research Center, Endocrinology and Metabolism Clinical Sciences Institute, Tehran University of Medical Sciences, Tehran, Iran; ^5^ Department of Electronic Engineering, Universitat Rovira i Virgili, Tarragona, Spain

**Keywords:** palladium catalyst, immobilized catalyst, mesoporous silica nanoparticles, Heck reaction, Suziki reaction

## Abstract

In this study, a novel catalyst is introduced based on the immobilization of palladium onto dipyrido (3,2-*a*:2′,3′-*c*) phenazine–modified mesoporous silica nanoparticles. The dipyrido (3,2-*a*:2′,3′-*c*) phenazine (Py_2_PZ) ligand is synthesized in a simple method from the reaction of 1,10-phenanthroline-5,6-dione and 3,4-diaminobenzoic acid as starting materials. The ligand is used to functionalize mesoporous silica nanoparticles (MSNs) and modify their surface chemistry for the immobilization of palladium. The palladium-immobilized dipyrido (3,2-*a*:2′,3′-*c*) phenazine–modified mesoporous silica nanoparticles (Pd@Py_2_PZ@MSNs) are synthesized and characterized by several characterization techniques, including TEM, SEM, FT-IR, TGA, ICP, XRD, and EDS analyses. After the careful characterization of Pd@Py_2_PZ@MSNs, the activity and efficiency of this catalyst is examined in carbon–carbon bond formation reactions. The results are advantageous in water and the products are obtained in high isolated yields. In addition, the catalyst showed very good reusability and did not show significant loss in activity after 10 sequential runs.

## Introduction

Carbon–carbon bond formation reactions, especially palladium-catalyzed ones, are significant reactions in organic chemistry. Among all the palladium-catalyzed carbon–carbon bond formation reactions, Heck and Suzuki reactions have attracted interests due to their high applications in various organic syntheses (Bahadorikhalili and Mahdavi, 2018; Christoffel and Ward, 2018; Mpungose et al., 2018; González-Sebastián and Morales-Morales, 2019; Sherwood et al., 2019; Tashrifi et al., 2019; Easson et al., 2020; Fusini et al., 2020). Regarding the unique advantages of Heck and Suzuki reactions, these reactions are used for the synthesis of several compounds with complex chemical structures (Bahadorikhalili et al., 2018a; Bahadorikhalili et al., 2020; Reza Hajipour and Khorsandi, 2020; Yao et al., 2020; Zhou et al., 2020). Therefore, several efforts have been focused on the introduction of novel catalysts with improved characteristics (Hajipour et al., 2018; Ma et al., 2018).

An interesting approach for designing novel catalysts for Heck and Suzuki reactions is the immobilization of palladium onto modified nanoparticles. This method enables both the advantages of homogenous and heterogenous catalysts. Several nanoparticles including nanosilica (Isfahani et al., 2013; Khalafi-Nezhad and Panahi, 2014), nitrogen-rich polymers (Targhan et al., 2020), chitosan (Kumari et al., 2017), magnetic iron oxide (Bahadorikhalili et al., 2015; Ma’mani et al., 2014), and graphene oxide (Kumari et al., 2019) have been used as a support for palladium catalysts. Mesoporous silica nanoparticles (MSNs) are an interesting ordered porous material made of silica with high surface area and unique physical and chemical properties. MSNs are chemically and physically stable and are compatible with biological systems (Mehmood et al., 2017; Narayan et al., 2018; Jeelani et al., 2020). MSNs have been used in various applications, including catalysis (Bahadorikhalili et al., 2018b; Sayahi et al., 2020), drug delivery (Bharti et al., 2015; Moreira et al., 2016), tissue engineering (Rosenholm et al., 2016), and pollutant removal from air and solutions (Li et al., 2016; Gao et al., 2017; Zarandi, 2019). Based on the high surface area and stability and ease of functionalization of MSNs, this family of nanoporous materials has extensively been used as a support for catalysts in various reactions (Han et al., 2006; Lee et al., 2009; Sarkar et al., 2019; Xu et al., 2019).

In spite of many reports on the immobilization of the catalytic agents onto modified nanoparticles, several of them hold a number of disadvantages. It is critical to investigate this field and synthesize catalysts which are efficient and selective and, in an ideal case, have the possibility of use in green solvents. As an interesting nanoparticle for the immobilization of the catalyst, MSNs are of high interest due to their advantages, including high surface area, high thermal stability, and high surface-active groups, which could be used for surface modifications. In this study, we introduce a novel palladium catalyst supported onto dipyrido (3,2-*a*:2′,3′-*c*) phenazine–modified MSNs. The catalytic activity of Pd@Py_2_PZ@MSNs is evaluated in Heck and Suzuki reactions. It should be noted that the palladium should be in the 0 oxidation state to catalyze the reaction (Zhang et al., 2006). Based on that, the catalyst should have been reduced to Pd (0) for efficiently catalyzing the reaction (Xiao et al., 2021).

## Results and Discussion

**SCHEME 1:**
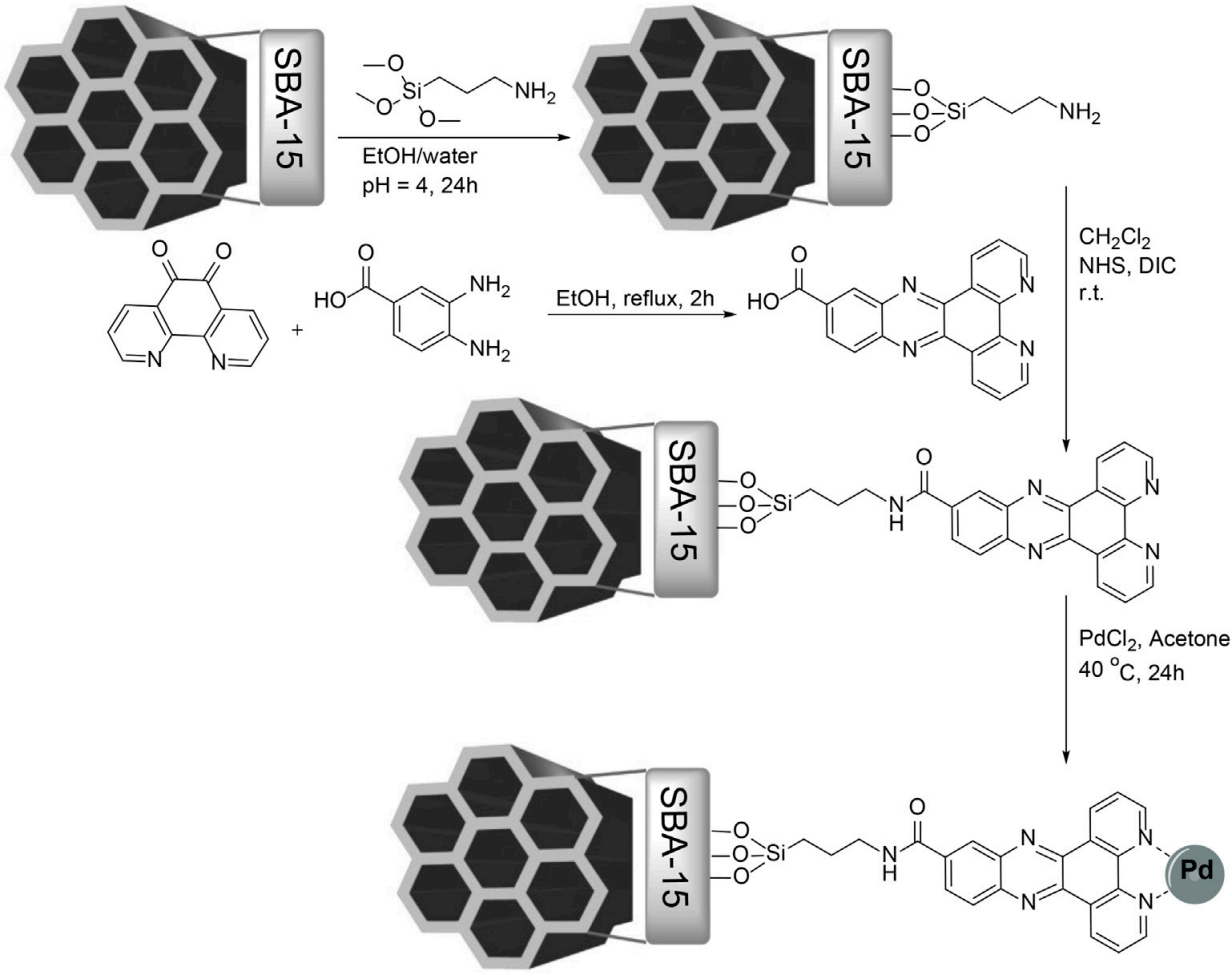
Synthesis of the Pd@Py_2_PZ@MSN catalyst.

In this study, SBA-15 MSNs were synthesized using the hydrothermal method and modified by (3-aminopropyl) methoxysilane. Dipyrido (3,2-*a*:2′,3′-*c*) phenazine (Py_2_PZ) was synthesized in a one-step reaction between 3,4-diaminobenzoic acid and 1,10-phenanthroline-5,6-dione, which enables it to react with amine groups on the surface of MSNs *via* an amidation reaction to yield Py_2_PZ@MSNs. The Pd@Py_2_PZ@MSN catalyst was prepared using Py_2_PZ@MSNs as a support, and it was fully characterized by various characterization techniques. The synthesis steps are presented in Scheme 1.

**FIGURE 1:**
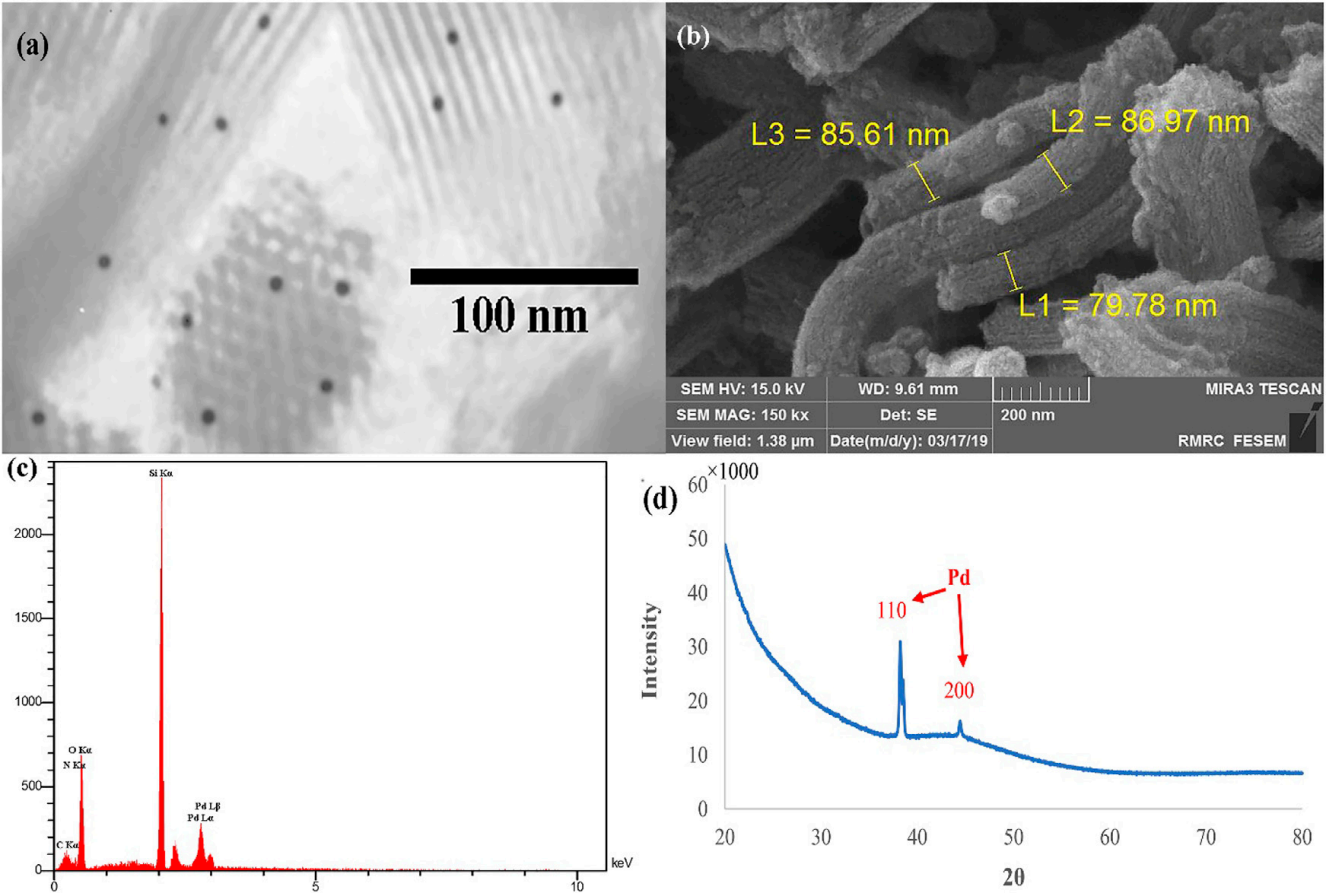
**(A)** TEM; **(B)** SEM; **(C)** EDS; and **(D)** XRD results of the Pd@Py_2_PZ@MSN catalyst.

Electron microscopy images of the Pd@Py_2_PZ@MSN catalyst are shown in Figure 1. The hexagonal meso-structure of the MSN backbone could be observed in the TEM image that Pd NPs appear as dark zones (Figure 1A). The rod-like structure of MSNs could clearly be observed in the SEM image (Figure 1B). In addition, the EDS analysis of the Pd@Py_2_PZ@MSN catalyst confirms the presence of Pd in the structure of the catalyst (Figure 1C). As seen in Figure 1D **,** the XRD pattern of the Pd@Py_2_PZ@MSN catalyst confirms that the crystallinity of the catalyst is similar to that of SBA-15 (Hu et al., 2016). In addition, the high-angle XRD confirms the presence of Pd NPs by two peaks of 110 and 200 planes at 2θ of 39.2 and 44.3°, respectively (Figure 1D).

**FIGURE 2:**
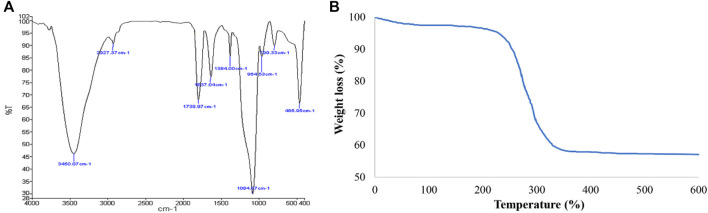
**(A)** FT-IR; and **(B)** TGA results of the Pd@Py_2_PZ@MSN catalyst.

To confirm the successful synthesis of Pd@Py_2_PZ@MSNs, the FT-IR spectra of nanomaterials are compared (Figure 2A). In the FT-IR spectra of Pd@Py_2_PZ@MSNs, a peak at 1084 cm^−1^ represents Si–O vibrations in the structure of the catalyst. The hydroxyl groups of the catalyst could be observed at 3450 cm^−1^, and the CH_2_ stretching vibration is presented at 2927 cm^−1^. The peak that appeared at 1739 cm^−1^ is attributed to the C=O bond found in the ester moiety in the structure of the Pd@Py_2_PZ@MSN catalyst.

The organic content in the structure of the catalyst was studied using TGA (Figure 2B). The TGA curve shows that the catalyst was thermally stable up to 250°C. A weight loss at the temperature of 250–350°C could be observed, which could be correlated to the degradation of organic moieties in the structure of the Pd@Py_2_PZ@MSN catalyst. The palladium content in the structure of the catalyst was measured using ICP analysis. The results showed that the Pd content in the Pd@Py_2_PZ@MSN catalyst was 0.12 mmol g^−1^.

The surface area and pore size of the Pd@Py2PZ@MSN catalyst were characterized using BET analysis and compared with those of MSNs. Based on the BET and nitrogen adsorption–desorption results, Py_2_PZ@MSNs have a high surface area of 612.37 m^2^ g^−1^. In addition, the pore diameter and pore volume of the synthesized nanoporous support are 5.14 nm and 0.72 cm^3^ g^−1^, respectively. The surface area, pore width, and pore volumes of MSNs are 655.37 m^2^ g^−1^, 5.75 nm, and 0.79 cm^3^ g^−1^, respectively. The decrease in surface area, pore width, and pore volume of MSNs by the synthesis of the catalyst is expected, while functionalization of the nanoparticles leads to such changes. The surface area and pore size results of MSNs and the Pd@Py_2_PZ@MSN catalyst are presented in Table 1.

**TABLE 1:** Surface area and pore size results of MSN and the Pd@Py_2_PZ@MSN catalyst.

	Surface area (m^2^.g^−1^)	Pore width (nm)	Pore volume (cm^3^.g^−1^)
MSN	655.65	5.75	0.79
Pd@Py_2_PZ@MSN	612.37	5.14	0.72

After the characterization of the Pd@Py_2_PZ@MSN catalyst, the efficiency of the catalyst was examined in carbon–carbon bond formation reactions. For this purpose, the Pd@Py_2_PZ@MSN catalyst was used in Heck and Suzuki reactions. Initially, the optimal reaction conditions were evaluated by performing the reaction under different reaction conditions including various solvents, bases, catalysts, and times of the reactions. To this end, the reaction of styrene and phenyl bromide was selected as a model reaction. The optimization results are presented in Table 2. According to the results, the best solvent for the reaction is water. However, the yields of the product were less in other solvents. Therefore, water was selected as the best solvent for the reaction. In addition, the results showed that the best yield of the product is obtained when the reaction is performed in the presence of 1 mol% of the catalyst. It should be noted that the presence of a base is critical for reaction performance. Therefore, the reaction was performed in the presence of several bases such as triethyl amine (TEA), NaOH, KOH, and pyridine. The results showed that TEA was the best base for the reaction. To study the role of the Pd@Py_2_PZ@MSN catalyst in the reaction, a blank run was performed by all the reaction components, except the catalyst. No product was obtained in the absence of the catalyst. Therefore, the optimized reaction conditions were selected to be water as the solvent and 1.5 equivalent of triethyl amine base in the presence of 1.0 mol% of the Pd@Py_2_PZ@MSN catalyst.

**TABLE 2:** Optimization of the reaction of styrene and phenyl bromide in the presence of the Pd@Py_2_PZ@MSN catalyst.

Entry	Solvent	Base (mol)	Catalyst (mol%)	Time (min)	Yield (%)
1	EtOH	TEA (1.5)	Pd@Py_2_PZ@MSN (1.0)	120	61
2	MeOH	TEA (1.5)	Pd@Py_2_PZ@MSN (1.0)	120	67
3	CH_2_Cl_2_	TEA (1.5)	Pd@Py_2_PZ@MSN (1.0)	120	47
4	DMF	TEA (1.5)	Pd@Py_2_PZ@MSN (1.0)	120	59
5	H_2_O	TEA (1.5)	Pd@Py_2_PZ@MSN (1.0)	120	94
6	H_2_O	NaOH (1.5)	Pd@Py_2_PZ@MSN (1.0)	120	80
7	H_2_O	KOH (1.5)	Pd@Py_2_PZ@MSN (1.0)	120	87
8	H_2_O	Pyridine (1.5)	Pd@Py_2_PZ@MSN (1.0)	120	69
9	H_2_O	K_2_CO_3_	Pd@Py_2_PZ@MSN (1.0)	120	77
10	H_2_O	No base	Pd@Py_2_PZ@MSN (1.0)	120	Trace
11	H_2_O	TEA (0.5)	Pd@Py_2_PZ@MSN (1.0)	120	55
12	H_2_O	TEA (1.0)	Pd@Py_2_PZ@MSN (1.0)	120	70
13	H_2_O	TEA (2.0)	Pd@Py_2_PZ@MSN (1.0)	120	94
14	H_2_O	TEA (1.5)	Pd@Py_2_PZ@MSN (0.5)	120	46
15	H_2_O	TEA (1.5)	Pd@Py_2_PZ@MSN (1.5)	120	94
16	H_2_O	TEA (1.5)	Pd@Py_2_PZ@MSN (1.0)	60	67
17	H_2_O	TEA (1.5)	Pd@Py_2_PZ@MSN (1.0)	150	94
18	H_2_O	TEA (1.5)	Pd/C (1.0)	120	56
19	H_2_O	TEA (1.5)	Pd(OAc)_2_ (1.0)	″	69
21	H_2_O	TEA (1.5)	PdCl_2_ (1.0)	″	61
22	H_2_O	TEA (1.5)	No catalyst	″	0

Reaction conditions: styrene (1 mmol); phenyl bromide (1 mmol); catalyst; solvent (5 ml); base; 25°C.

To rule out the presence of leached and homogenous Pd in the reaction mixture, in a run, the reaction was performed under the optimal reaction condition. After 60 min, the conversion of the substrates to the product measured by GC was 67%. Then, the catalyst was separated from the reaction mixture, and the filtrate was stirred under the same conditions for more 60 min. The analysis of the product by GC showed that no more product has been obtained. These observations confirmed that the Pd catalyst worked in the heterogenous phase and the presence of the Pd@Py_2_PZ@MSN catalyst was necessary for reaction performance.

Based on the optimized conditions, the scope and generality of the Pd@Py_2_PZ@MSN catalyst were studied in Heck and Suzuki reactions. The structures and yields of the products are presented in Table 3. The high efficiency of the Pd@Py_2_PZ@MSN catalyst in the mentioned reaction could clearly be observed. The catalyst is highly active in both Heck and Suzuki reactions, and advantageous results were obtained. All substrates with electron donating and electron withdrawing functionalities have given the desired products in high isolated yields. For example, entries 4–9, 18–22, and 26–28 with phenyl halides bearing electron-donating functionalities have provided the products in 84–93% yields. In addition, phenyl halides bearing electron-withdrawing functionalities (entries 10–14, 22–23, and 29–31) have successfully participated in the reaction and have given the products in 88–99% yields. In addition, a reaction was performed with 10 mmol of the substrates to evaluate the possible scale up of the reaction. The results were advantageous, and high yields of the products were obtained in the scale-up reactions. To study the possibility of the use of the catalyst in industrial applications, scale-up reaction was performed under optimized conditions. The large-scale reaction of iodobenzene with styrene and phenylboronic acid has led to 85 and 83% yields, respectively.

**TABLE 3:** Scope and generality of the Pd@Py_2_PZ@MSN catalyst in Heck and Suzuki reactions^a^.
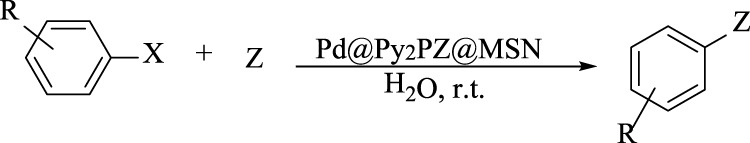

Entry	R	Z	X	Time (min)	Yield (%)	TON^b^	TOF^c^
1	H	Styrene	Br	120	94	9400	4700
2	H	Styrene	Cl	150	79	7900	3160
3	H	Styrene	I	120	96	9600	4800
4	4–Me	Styrene	Br	120	90	9000	4500
5	4–Me	Styrene	Cl	150	75	7500	3000
6	4–Me	Styrene	I	120	94	9400	4700
7	4–OMe	Styrene	Br	120	91	9100	4550
8	4–OMe	Styrene	I	120	93	9300	4650
9	4–NMe_2_	Styrene	Br	120	93	9300	4650
10	4–Cl	Styrene	Br	120	96	9600	4800
11	4–Cl	Styrene	Cl	150	70	8000	3200
12	4–CN	Styrene	Br	120	94	9400	4700
13	4–CN	Styrene	Cl	150	78	8800	3520
14	4–CHO	Styrene	Br	120	93	9300	4650
15	H	butyl acrylate	Br	120	96	9600	4800
16	H	butyl acrylate	Cl	150	82	8200	3280
17	H	butyl acrylate	I	90	96	9600	6400
18	4–Me	butyl acrylate	Br	120	89	8900	4450
19	4–Me	butyl acrylate	Cl	150	75	7500	3000
20	4–OMe	butyl acrylate	Br	120	91	9100	4550
21	4–OMe	butyl acrylate	Cl	150	76	7600	3040
22	4–CN	butyl acrylate	Br	120	94	9400	4700
23	4–CN	butyl acrylate	Cl	150	89	8900	3560
24	H	Ph(BOH)_2_	Br	120	92	9200	4600
25	H	Ph(BOH)_2_	I	150	97	9700	3880
26	4–Me	Ph(BOH)_2_	Br	120	88	8800	4400
27	4–Me	Ph(BOH)_2_	I	150	92	9200	3680
28	4–OMe	Ph(BOH)_2_	Br	120	84	8400	4200
29	4–Cl	Ph(BOH)_2_	Br	120	93	9300	4650
30	4–CN	Ph(BOH)_2_	Br	120	95	9500	4750
31	4–CN	Ph(BOH)_2_	I	150	99	9900	3960
32^d^	H	Styrene	I	120	85	8500	4250
33^d^	H	Ph(BOH)_2_	I	120	83	8300	4150

a Reaction conditions: styrene, butyl acrylate, or phenylboronic acid (1 mmol); phenyl halide (1 mmol); Pd@Py_2_PZ@MSN catalyst (1 mol%); H_2_O (5 ml); TEA (1.5 mmol); 25°C.

b TON is calculated as the number of moles of the substrate converted to the product divided by the number moles of palladium catalyst.

c TOF is defined as TON h^−1^.

d Scale-up reactions: styrene or phenylboronic acid (10 mmol); phenyl bromide (10 mmol); Pd@Py_2_PZ@MSN catalyst (1 mol%); H_2_O (50 ml); TEA (15 mmol); 25°C.

**SCHEME 2:**
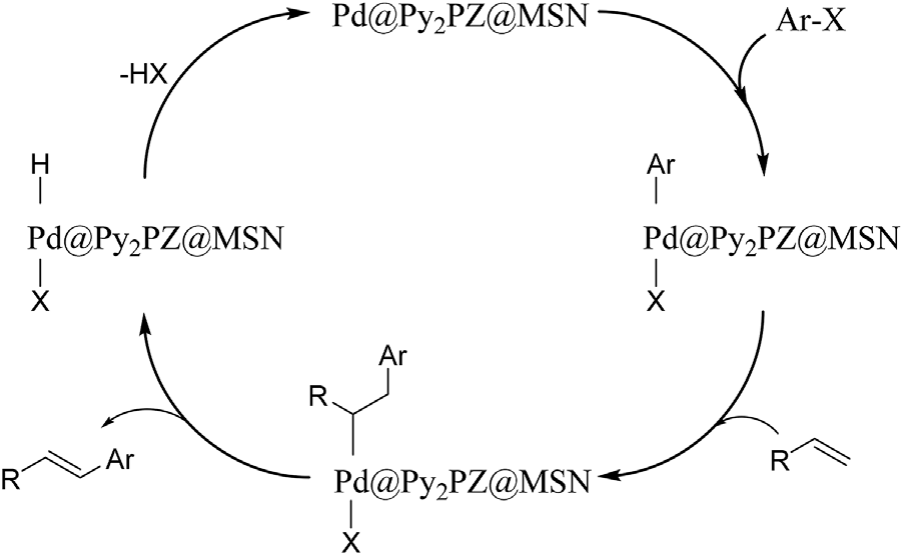
Proposed mechanism for Pd@Py2PZ@MSN catalyzed carbon–carbon bond formation.

A proposed mechanism was suggested, which is presented in Scheme 2. Based on the mechanism, the presence of the catalyst is critical for reaction performance. In the first step of the reaction, the catalyst activates the Ar–X bond, followed by the addition of the alkene. In the next step, the product is obtained, and the catalyst is ready for the next cycle of the reaction by the elimination of H–X.

**FIGURE 3:**
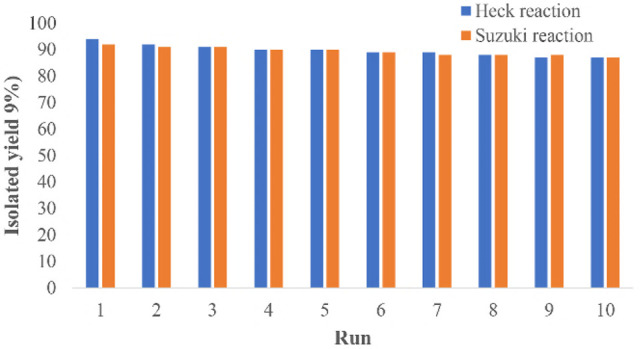
Recovery results of the Pd@Py_2_PZ@MSN catalyst.

A great advantage of the Pd@Py_2_PZ@MSN catalyst is its reusability. For this purpose, the reaction was repeated for 10 sequential reactions, and the obtained results are presented in Figure 3. It could be seen that in both Heck and Suzuki reactions, no big loss in the activity of the catalyst is observed. It is significant to study the possible leaching of palladium from the catalyst. For studying the leaching of palladium from the Pd@Py_2_PZ@MSN catalyst, a reaction was performed under the optimized reaction conditions. However, after 20 min when the reaction has not been completed, the catalyst was isolated from the reaction mixture. The reaction was continued without the catalyst for more than 3 h and based on GC analysis, no performance was observed in the reaction performance. In addition, the filtrate was studied by ICP, which did not show any leaching of palladium from the catalyst.

**FIGURE 4:**
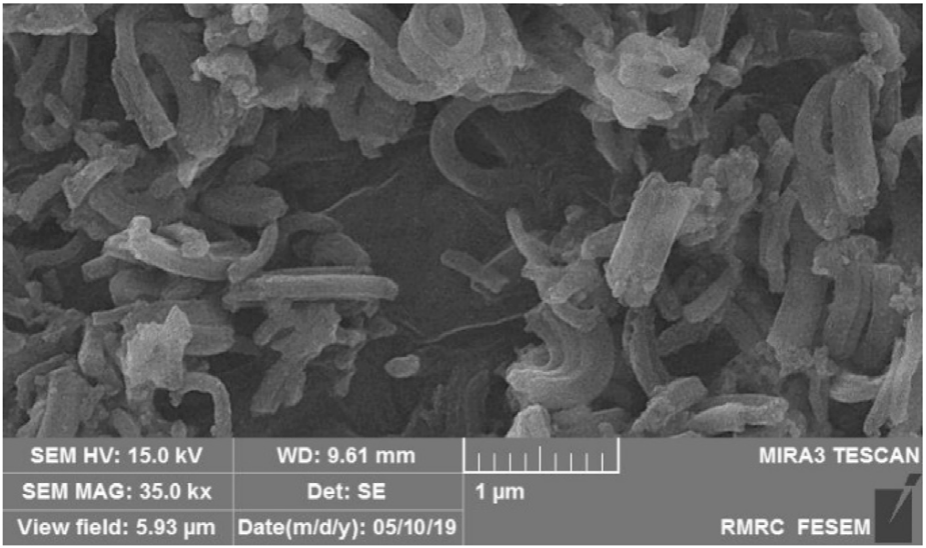
SEM image of the recovered Pd@Py_2_PZ@MSN catalyst.

To show the structural stability of the catalyst during the reaction conditions, the catalyst was separated after the 5^th^ reaction run and washed with water and ethanol. Then, the dried 5^th^ re-used Pd@Py_2_PZ@MSN catalyst was characterized by SEM and BET analysis. The results are presented in Figure 4; Table 4, respectively. Comparing the results of the recovered catalyst with the fresh ones confirm the good stability of the catalyst during the reactions.

**TABLE 4:** Surface area and pore size results of the recovered Pd@Py_2_PZ@MSN catalyst.

Surface area (m^2^.g^−1^)	Pore width (nm)	Pore volume (cm^3^.g^−1^)
607.21	5.09	0.70

To better study the activity of the catalyst, Pd@Py_2_PZ@MSNs were compared with the previously reported catalysts. Table 5 presents the catalysts, reaction conditions, and isolated yields of the reactions for the reaction of styrene and bromobenzene. According to the results, the best performance is observed for Pd@Py_2_PZ@MSNs, and the reaction is performed under mild reaction conditions.

**TABLE 5:** Comparison of the catalytic activity of Pd@Py_2_PZ@MSN with the previously reported one.

Entry	Catalyst	Conditions	Time (h)	Yields^a^ (%)^ref^
1	Pd/TiO_2_ NP	DMF, Et_3_N, 140°C	10	93 Nasrollahzadeh et al. (2014)
2	Pd/SMNPs-DF	Solvent free, DABCO, 140°C	0.9	93 Zolfigol et al. (2014)
3	CO-NHC@MWCNTs	PEG, Li_2_CO_3_, 80°C	10	67 Hajipour and Khorsandi, (2016)
4	Pd/MPCS-TI	DMF/H_2_O, Et_3_N, 110°C	4	93 Movassagh and Rezaei, (2015)
5	Pd/BIP-silica-Fe_2_O_3_	DMF, Et_3_N, 100°C	3.5	82 Sobhani et al. (2015)
6	Pd/Guanidine/graphene	EtOH/H_2_O, K_2_CO_3_, r.t.	2	92 Ma’mani et al. (2014)
7	This work	H_2_O, TEA, 25°C	1	94

a Isolated yields.

## Experimental

### General Remarks

All the chemicals, reagents, and solvents were purchased from Merck, Germany, and Sigma, Germany. TEM images were recorded using a CM 10 Philips instrument. ^1^H (500 MHz) spectra were recorded using a Bruker Advance spectrometer in DMSO and d_6_ solution with tetramethylsilane (TMS) as an internal standard. A TESCAN T3000 instrument was used to record the SEM images. An OPTIMA7300DV ICP analyzer was used for analyzing the palladium content of the catalyst. The FT-IR spectra were recorded on a Nicolet Magna FT-IR 550 spectrophotometer using potassium bromide disks. BET of the samples was analyzed using an ASAPTM micromeritics 2020 instrument. The particle size was measured using the HORIBA SZ100-Z DLS instrument. The thermogravimetric analyzer (TGA) at a heating rate of 10°C min^−1^ over the temperature range of 40–600°C under nitrogen atmosphere was used.

### Synthesis of the Py_2_PZ Ligand

1,10-phenanthroline-5,6-dione (1.05 g, 5 mmol) and 3,4-diaminobenzoic acid (0.76 g, 5 mmol) were dissolved in ethanol and refluxed for 2 h. The reaction progress was monitored by TLC monitoring. After the reaction was completed, the solvent was evaporated and the product was purified by recrystallization from ethanol.

### Synthesis of the Pd@Py_2_PZ@MSN Catalyst

SBA-15 was synthesized according to the previously reported method (Chaudhary and Sharma, 2017). A mixture containing MSNs (0.5 g) in ethanol/deionized water (1:2, 30 ml) and HCl (pH = 4) was prepared and sonicated for 30 min. A solution of (3-aminopropyl) methoxysilane (1 g) in ethanol (30 ml) was prepared and added drop-wise to the aforementioned mixture by vigorous stirring. The reaction mixture was stirred for 24 h, and then the product was separated by filtration and washed with deionized water and ethanol. The product was dried at 70°C in vacuum for 12 h to obtain amine-modified mesoporous SBA-15.

A mixture of amine-modified mesoporous SBA-15 (0.5 g) in dichloromethane (75 ml) was sonicated for 30 min. Then, dipyrido (3,2-*a*:2′,3′-*c*) phenazine-11-carboxylic acid (1.6 g, 5 mmol) in dichloromethane (25 ml) was added and 1-ethyl-3-(3-dimethylaminopropyl) carbodiimide (5 mmol, 1 eq) and *N*-hydroxysuccinnimide (5.5 mmol, 1.1 eq) were added. The reaction mixture was stirred overnight at room temperature. The product was separated from the reaction mixture and washed with ethanol and dried for 12 h in a vacuum oven.

The solid support (0.5 g) was added to dry acetone and sonicated for 30 min. Then, PdCl_2_ (1 mmol) was added, and the reaction mixture was stirred at 40°C under an inert atmosphere for 24 h. The Pd@Py_2_PZ@MSN catalyst was separated from the reaction mixture by centrifugation, washed thoroughly with EtOH and diethyl ether, and dried under vacuum at room temperature for 12 h.

### General Procedure for the Carbon–Carbon Bond Formation Reactions

Phenyl halide (1 mmol), alkene (1.1 mmol, in case of Heck reaction), phenylboronic acid (1.0 mmol, in case of Suzuki reaction), triethyl amine (1.5 mmol), and the Pd@Py_2_PZ@MSN catalyst (1.0 mol%) were added into a flask containing H_2_O (5 ml) and stirred at room temperature. When the TLC monitoring showed reaction completion, the catalyst was separated from the reaction mixture and washed with water and EtOH. The product was extracted with ethyl acetate. The organic phase was collected and dried over sodium sulfate. The solvent was evaporated, and the product was purified by column chromatography using *n*-hexane:ethyl acetate (6:1) as the eluent to obtain the pure product.

### Recovery of the Nanocatalyst

The reaction of phenyl bromide (1 mmol) and styrene (1.1 mmol) was selected as a model reaction and was performed in the presence of the Pd@Py_2_PZ@MSN catalyst under optimal reaction conditions. After the reaction was completed, the catalyst was separated from the reaction mixture and washed with water and used in the next reaction under the same conditions. The reactions were performed for 10 sequential runs.

## Conclusion

In conclusion, Pd@Py_2_PZ@MSN as a novel catalyst was designed, synthesized, and characterized based on the immobilization of palladium onto the modified SBA-15 MSNs. The catalyst was characterized using several methods, and the structure of the catalyst was confirmed. The activity of the Pd@Py_2_PZ@MSN catalyst was evaluated in Heck and Suzuki reactions. The catalyst showed the best activity in water as the solvent in the presence of triethyl amine base. Several substrates with different functionalities were used and, in all cases, the desired compounds were synthesized in good isolated yields. As an advantage, the catalyst showed very good reusability after 10 sequential runs.

## Data Availability

The raw data supporting the conclusions of this article will be made available by the authors, without undue reservation.
